# Development of a Visual RPA-CRISPR/Cas12a Assay Targeting *Bcsp31* for Rapid Detection of *Brucella* spp.

**DOI:** 10.3390/vetsci13080820

**Published:** 2026-08-17

**Authors:** Shuairan Zhang, Yu Zhang, Cai Yin, Xiaona Zang, Minjing Jin, Yawen Wu, Taotao Bai, Long Ma, Yong Shi, Zhixin Li, Xiaoliang Wang

**Affiliations:** 1 College of Animal Science and Technology, Ningxia University, Yinchuan 750021, China; 18235177611@163.com (S.Z.);; 2 Ningxia Hui Autonomous Region Animal Disease Prevention and Control Center, Yinchuan 750001, China; 3 College of Life Science and Technology, Ningxia Vocational and Technical University, Yinchuan 750021, China

**Keywords:** *Brucella* spp., brucellosis, bcsp31, recombinase polymerase amplification, CRISPR/Cas12a, visual detection, qPCR

## Abstract

**Simple Summary:**

Brucellosis is an important zoonotic disease affecting sheep, goats, cattle, and humans. Rapid screening methods that require only simple equipment are useful for veterinary surveillance, especially in primary laboratories and field settings. In this study, a visual nucleic acid detection method combining recombinase polymerase amplification (RPA) with CRISPR/Cas12a was developed for *Brucella* spp. detection. The assay targets the conserved *bcsp31* gene and can be judged by fluorescence after amplification and Cas12a-mediated signal generation. The optimized assay detected low-copy plasmid templates and showed no cross-reactivity with several common sheep-associated pathogens tested in this study. In 56 sheep nasal swab samples collected during routine surveillance, the method showed high diagnostic agreement with qPCR. These results suggest that the assay may provide a convenient tool for rapid preliminary screening of *Brucella* spp. in veterinary surveillance.

**Abstract:**

To establish a rapid nucleic acid detection method for field screening of *Brucella* spp., a visual assay based on recombinase polymerase amplification (RPA) combined with CRISPR/Cas12a was developed. Four pairs of RPA primers and two crRNAs were designed according to the conserved *Brucella bcsp31* sequence. The optimal RPA primer–crRNA combination was screened using agarose gel electrophoresis and fluorescence readout, and the concentrations of Cas12a protein, crRNA, and ssDNA fluorescent reporter were optimized. RPA primer pair 2 showed the best amplification performance, and RPA2-crRNA1 was selected as the optimal combination for the assay. The final reaction conditions were 200 nM Cas12a protein, 200 nM crRNA, and 250 nM ssDNA fluorescent reporter. The limit of detection of the established method was 2.47 × 10^0^ copies/μL. No cross-reactivity was observed with *Mycoplasma capricolum* subsp. *capripneumoniae*, *Chlamydia abortus*, *Chlamydia psittaci*, or *Salmonella enterica* serovar Typhimurium. Among 56 clinical sheep nasal swab samples collected during routine surveillance, qPCR identified 22 positive and 34 negative samples, while the RPA-CRISPR/Cas12a assay identified 20 positive and 36 negative samples. The positive agreement, negative agreement, overall agreement, positive predictive value, negative predictive value, and Cohen’s kappa value were 90.91%, 100.00%, 96.43%, 100.00%, 94.44%, and 0.924, respectively. These findings provide preliminary evidence that the developed bcsp31-targeted RPA-CRISPR/Cas12a assay is rapid and visually interpretable and may be useful for preliminary *Brucella* spp. screening in routine veterinary surveillance settings. Larger-scale validation using different sample types and samples from different regions is still required.

## 1. Introduction

Brucellosis is a widespread zoonotic disease in livestock farms and pastoral areas. It threatens animal production and public health and can cause substantial economic losses. Although multiple prevention, monitoring, and eradication policies have been implemented, brucellosis has not been completely eliminated and continues to circulate in some regions due to animal movement, farming patterns, and other epidemiological factors [1,2,3]. Early detection of infected animals and rapid screening of suspected clinical samples are therefore important for controlling disease transmission.

Several diagnostic approaches are available for brucellosis. Bacteriological isolation is regarded as a direct method for identifying the pathogen, but it is time-consuming, requires biosafety conditions, and is more suitable for samples containing sufficient viable organisms. Serological tests, such as the Rose Bengal plate test, are convenient for large-scale screening; however, they detect host antibody responses rather than pathogen nucleic acids and can be affected by infection stage, vaccination history, and cross-reactions [4,5]. PCR and qPCR are important molecular tools for *Brucella* detection, but their performance can be influenced by target selection, sample type, DNA extraction quality, and standardization of protocols [5,6]. qPCR also requires specialized instruments and trained personnel. Therefore, a rapid, simple, and visually interpretable nucleic acid detection method would be valuable for primary veterinary laboratories and field surveillance.

Recombinase polymerase amplification (RPA) is an isothermal nucleic acid amplification technique that amplifies target sequences under relatively mild constant-temperature conditions, without requiring a thermal cycler [7,8,9]. RPA has the advantages of rapid reaction, simple operation, and high sensitivity, and its amplification products can be analyzed by agarose gel electrophoresis, fluorescence detection, lateral flow strips, or downstream CRISPR/Cas-based detection systems [8,9].

Clustered regularly interspaced short palindromic repeats and CRISPR-associated proteins (CRISPR/Cas) constitute an adaptive immune system in prokaryotes [10,11]. Cas12a can be guided by crRNA to recognize specific DNA sequences and, upon activation, cleave single-stranded DNA reporters to generate detectable signals. In recent years, RPA combined with CRISPR/Cas12a has been widely explored for rapid visual detection of pathogens [12]. RPA enriches low-copy targets within a short time, whereas Cas12a provides an additional sequence-recognition step and improves the specificity of the readout.

Although RPA-CRISPR/Cas12a-based strategies have shown potential for rapid nucleic acid detection, further methodological evaluation is still needed for different target designs, primer–crRNA combinations, visual readout formats, and routine surveillance sample types. In field or primary veterinary settings where rapid testing and minimal equipment dependence are required, a visual RPA-CRISPR/Cas12a assay may help veterinarians and diagnostic personnel rapidly identify suspected *Brucella*-positive samples and make timely preliminary screening decisions. In particular, limited information is available regarding the application of a *bcsp31*-targeted visual RPA-CRISPR/Cas12a workflow to sheep nasal swab samples collected during routine brucellosis surveillance.

## 2. Materials and Methods

### 2.1. Reagents, Instruments, and Clinical Samples

The conserved *bcsp31* sequence of *Brucella* spp. (GenBank accession No. M20404.1) was used as the target sequence. A pUC19-*bcsp31* positive plasmid was synthesized by Tsingke Biotechnology Co., Ltd., Beijing, China. A total of 56 sheep nasal swab samples were randomly selected from samples collected during the routine second-quarter brucellosis surveillance and random monitoring program in 2026 in Ningxia Hui Autonomous Region, China. The samples were obtained from sheep farms in Wuzhong City, Ningxia Hui Autonomous Region, and were used for preliminary evaluation of the established RPA-CRISPR/Cas12a assay. The samples were collected as part of routine veterinary surveillance according to local surveillance arrangements rather than specifically for this study. According to the available farm vaccination records, the sampled animals had received foot-and-mouth disease vaccine on 30 October 2025, supplied by Jinyu Technology, and a peste des petits ruminants bivalent vaccine, supplied by Huapai Bio-engineering Group Co., Ltd., Chengdu, Sichuan, China, before sample collection. No Brucella vaccine was administered to the sampled animals according to the available vaccination records. Therefore, Brucella vaccine strains or vaccine-derived Brucella nucleic acids were not expected to affect the interpretation of the qPCR or RPA-CRISPR/Cas12a results in this study. No additional animal experiment, blood collection, or invasive intervention was performed specifically for this study. The swabs were transported to the laboratory under cold-chain conditions and used for nucleic acid extraction and assay evaluation. Table 1 lists the main reagents and their manufacturers, while Table 2 lists the main instruments.

### 2.2. Primer and CrRNA Design

Four pairs of RPA primers were designed from the *Brucella bcsp31* sequence (GenBank accession No. M20404.1) using Primer5 software and synthesized by Tsingke Biotechnology Co., Ltd. Candidate RPA primers were selected according to the general principles of RPA primer design, including a primer length of approximately 30–35 nucleotides, GC content of 40–60%, and an expected amplicon size of 100–400 bp. The predicted risks of hairpin structures, primer dimers, cross-dimers, and false priming were also considered using Primer5. Four primer pairs with acceptable predicted parameters were selected for subsequent experimental screening. Two candidate crRNAs were designed using the online tool ezassay.com. Candidate crRNA spacer sequences located within the RPA amplicons and with relatively high evaluation scores were selected, and a fixed scaffold sequence was added before each spacer. crRNAs were synthesized by General Biosystems (Anhui) Co., Ltd., China, Anhui, Chuzhou. The sequences are shown in Table 3.

### 2.3. Establishment of the RPA Reaction

The synthesized pUC19-*bcsp31* plasmid was used as the template for RPA. For each 25 μL reaction, 5 μL of template was added to the tube containing the RPA lyophilized pellet. Then, 1 μL each of forward and reverse primer (10 μmol/L), 1.25 μL of Mg^2+^ buffer, and nuclease-free water were added to a final volume of 25 μL. According to the manufacturer’s instructions for the RPA kit, the recommended incubation condition was 42 °C for 20 min. In this study, the incubation time was further evaluated, and incubation at 42 °C for 30 min produced clearer amplification and stronger downstream fluorescence signals than the recommended 20 min reaction. Therefore, the reaction mixture was mixed by gentle inversion, briefly centrifuged, and incubated at 42 °C for 30 min in a water bath for subsequent experiments. After amplification, products were purified using a DNA purification kit and analyzed by agarose gel electrophoresis.

To screen RPA primers, the pUC19-*bcsp31* plasmid was used as the template and the four primer pairs were tested under the same reaction conditions. The amplification products were analyzed using 1% agarose gel electrophoresis, and primer pairs producing clear bands were further evaluated in combination with crRNAs.

### 2.4. Screening of RPA Primer–crRNA Combinations

Purified RPA products were used as templates for the CRISPR/Cas12a reaction. The reaction system is shown in Table 4. The mixture was vortexed gently, briefly centrifuged, and incubated at 37 °C for 20 min. Fluorescence signals were collected every 30 s, and fluorescence was observed under ultraviolet light at the end of the reaction.

The four RPA products were separately combined with crRNA1 and crRNA2 to generate eight combinations: RPA1-crRNA1, RPA2-crRNA1, RPA3-crRNA1, RPA4-crRNA1, RPA1-crRNA2, RPA2-crRNA2, RPA3-crRNA2, and RPA4-crRNA2. Fluorescence intensity was compared under identical CRISPR/Cas12a reaction conditions, and the best-performing RPA primer–crRNA combination was selected for further optimization.

### 2.5. Optimization of Cas12a, crRNA, and ssDNA Reporter Concentrations

To determine suitable working concentrations of Cas12a protein and crRNA, concentrations of 0, 50, 100, 150, 200, and 250 nM were tested in an orthogonal combination design. After reaction, fluorescence was observed under ultraviolet light and endpoint fluorescence values were measured. The appropriate concentrations of Cas12a protein and crRNA were determined based on fluorescence intensity, background signal, and reagent consumption.

After the optimal concentrations of Cas12a protein and crRNA were determined, ssDNA fluorescent reporter concentrations of 0, 50, 100, 150, 200, and 250 nM were tested. The fluorescence signal at each reporter concentration was compared, and the final reporter concentration was selected based on visual fluorescence intensity, endpoint fluorescence values, and cost considerations.

### 2.6. Analytical Sensitivity

After extraction of the pUC19-*bcsp31* plasmid, the plasmid DNA concentration was measured as 100 ng/μL. The total recombinant plasmid length was 3695 bp, including the 2686 bp pUC19 vector and the 1009 bp insert. The plasmid copy number was calculated using the formula copies/μL = (C × 6.02 × 10^23^)/(L × 660 × 10^9^), where C is the plasmid concentration in ng/μL and L is the plasmid length in bp. The initial copy number of the pUC19-*bcsp31* plasmid was calculated to be approximately 2.47 × 10^10^ copies/μL. The plasmid was serially diluted 10-fold, and concentrations from 2.47 × 10^5^ to 2.47 × 10^−1^ copies/μL were used for the sensitivity test. Nuclease-free water was used as the negative control. Each concentration was tested using the optimized RPA-CRISPR/Cas12a system.

For endpoint fluorescence analysis, the positive cutoff was calculated as the mean fluorescence value of the negative control plus three standard deviations (meanNC + 3SDNC). The lowest template concentration that exceeded the cutoff and produced stable visual fluorescence was defined as the limit of detection.

### 2.7. Specificity Evaluation

The pUC19-*bcsp31* plasmid was used as the positive control, and nuclease-free water was used as the negative control. Nucleic acids extracted from positive clinical materials of *Mycoplasma capricolum* subsp. *capripneumoniae*, *Chlamydia abortus*, *Chlamydia psittaci*, and *Salmonella enterica* serovar Typhimurium were used as non-target templates. These four non-target pathogens were selected because they are clinically relevant pathogens considered in the differential diagnosis of sheep abortion and because positive clinical materials for these organisms were available in Ningxia during the study period. Due to the availability of positive clinical materials during routine surveillance, the specificity panel did not include phylogenetically closer organisms or bacteria known to be associated with diagnostic cross-reactions in brucellosis, such as *Ochrobactrum* spp. and *Yersinia enterocolitica* O:9. This limitation was considered when interpreting the specificity results. All templates were tested using the optimized RPA-CRISPR/Cas12a reaction conditions. Fluorescence was observed under ultraviolet light, and endpoint fluorescence values were recorded to evaluate specificity.

### 2.8. Clinical Sample Validation and qPCR Comparator Testing

A total of 56 sheep nasal swab samples obtained during routine brucellosis surveillance were tested using the optimized RPA-CRISPR/Cas12a assay. Genomic DNA was extracted using a commercial DNA/RNA extraction kit according to the manufacturer’s instructions. qPCR was performed using a commercial Brucella Real-time PCR Test Kit (Qingdao Lijian, Qingdao, China) according to the manufacturer’s instructions and was used as the comparator assay for diagnostic agreement analysis. The kit detects Brucella nucleic acid through the FAM fluorescence channel. The primer and probe reagents were supplied as premixed components in the kit, and the exact target gene, primer sequences, and probe sequence were not disclosed by the manufacturer. For each reaction, 20 μL of PCR reaction mixture was added to the PCR tube, followed by 5 μL of extracted sample DNA, positive control, or negative control, giving a final reaction volume of 25 μL. The qPCR amplification program was as follows: 37 °C for 2 min; 95 °C for 20 s; followed by 40 cycles of 95 °C for 10 s and 60 °C for 30 s, with FAM fluorescence signal collected at 60 °C. The run was considered valid only when the positive control showed a Ct value < 35 and the negative control showed no Ct value. According to the manufacturer’s interpretation criteria, samples with a Ct value < 37 in the FAM channel were considered positive for Brucella nucleic acid, samples with 37 ≤ Ct ≤ 40 were considered suspected positive and were retested when necessary, and samples with no Ct value were considered negative. For suspected samples, three repeated reactions were performed; if any reaction showed a Ct value ≤ 40, the sample was considered positive, whereas samples with no Ct value in all three repeated reactions were considered negative. Positive and negative qPCR results were interpreted according to the laboratory diagnostic criteria. The positive agreement, negative agreement, overall agreement, positive predictive value, negative predictive value, and Cohen’s kappa value were calculated to assess agreement between the RPA-CRISPR/Cas12a assay and qPCR.

### 2.9. Statistical Analysis

Fluorescence data are presented as the mean ± standard deviation (SD) of three replicates. The positive cutoff was defined as meanNC + 3SDNC. Endpoint fluorescence values were processed and statistically analyzed using GraphPad Prism version 9.5.0 (GraphPad Software, San Diego, CA, USA). For comparisons among multiple experimental groups in the RPA primer–crRNA screening experiment, one-way analysis of variance (one-way ANOVA) followed by Tukey’s multiple-comparison test was performed. A *p* value < 0.05 was considered statistically significant. For the optimization of Cas12a/crRNA and ssDNA reporter concentrations, endpoint fluorescence values were presented as mean ± SD, and the optimized conditions were selected based on endpoint fluorescence intensity, visual fluorescence readout, background signal, and reagent consumption. Clinical diagnostic agreement with qPCR was assessed using positive percent agreement (PPA), negative percent agreement (NPA), overall percent agreement (OPA), positive predictive value (PPV), negative predictive value (NPV), and Cohen’s kappa coefficient. The 95% confidence intervals (95% CIs) for PPA, NPA, OPA, PPV, and NPV were calculated using the exact binomial method. Cohen’s kappa coefficient and its 95% CI were calculated from the 2 × 2 contingency table.

### 2.10. Use of Generative AI Tools

During manuscript preparation and revision, ChatGPT 5.5 was used only for English translation and language polishing. The tool was not used for experimental design, data generation, statistical analysis, result interpretation, reference selection, or scientific conclusion development. All AI-assisted text was reviewed, revised, and verified by the authors.

## 3. Results

### 3.1. Screening of RPA Primers

**Figure 1:**
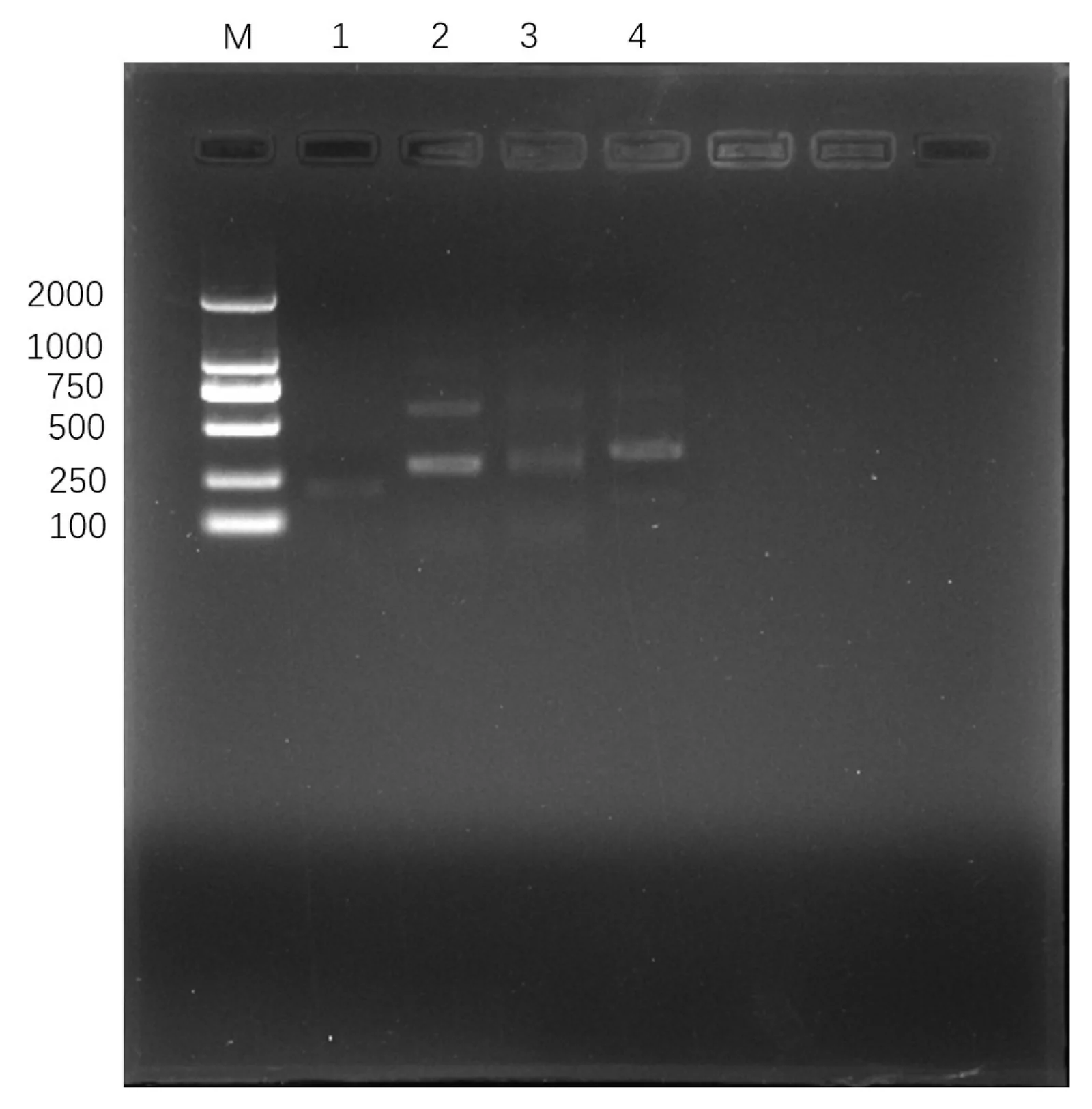
Screening results of RPA primers. M, marker; lane 1, amplification product of the RPA-F1/R1 primer pair; lane 2, amplification product of the RPA-F2/R2 primer pair; lane 3, amplification product of the RPA-F3/R3 primer pair; lane 4, amplification product of the RPA-F4/R4 primer pair.

The four RPA primer pairs were tested using the pUC19-*bcsp31* plasmid as the template. As shown in Figure 1, all four primer pairs produced amplification products, but the amplification efficiency differed among primer pairs. RPA2 produced the clearest and brightest band, suggesting better amplification performance than the other primer pairs. Because both RPA efficiency and Cas12a-based recognition can affect the final detection signal, all RPA products were further evaluated in combination with the two crRNAs.

### 3.2. Screening Result of RPA Primer–crRNA Combinations

**Figure 2:**
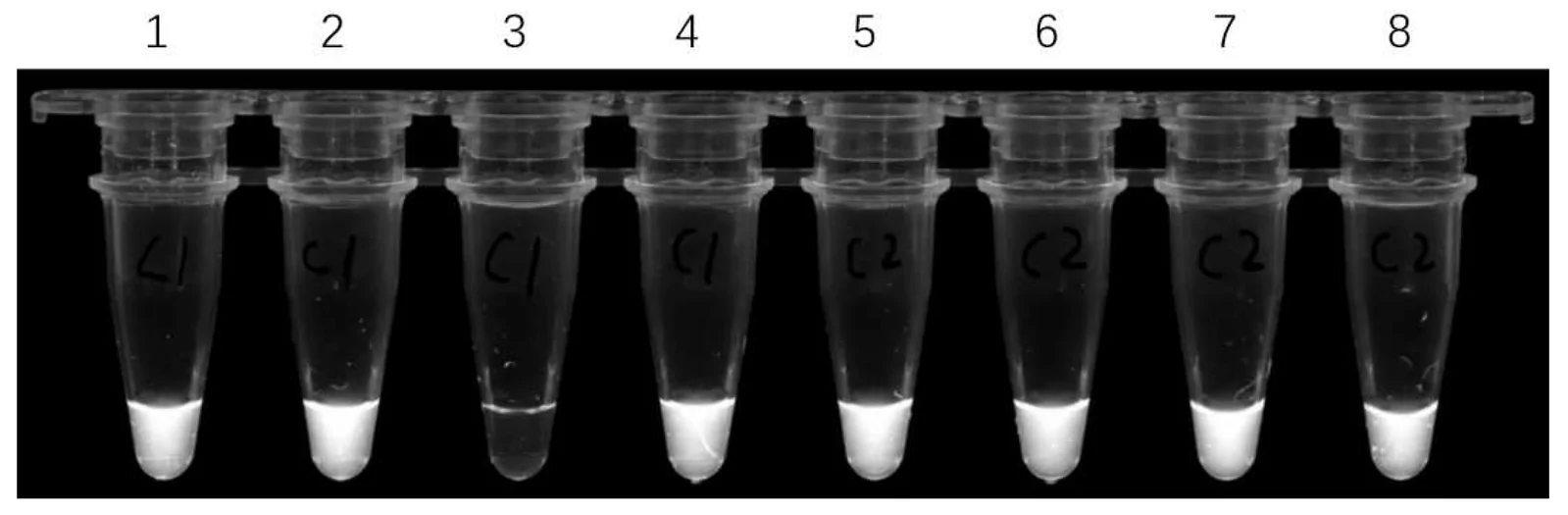
Fluorescence image for screening RPA and crRNA combinations. 1, RPA1-crRNA1; 2, RPA2-crRNA1; 3, RPA3-crRNA1; 4, RPA4-crRNA1; 5, RPA1-crRNA2; 6, RPA2-crRNA2; 7, RPA3-crRNA2; 8, RPA4-crRNA2.

**Figure 3:**
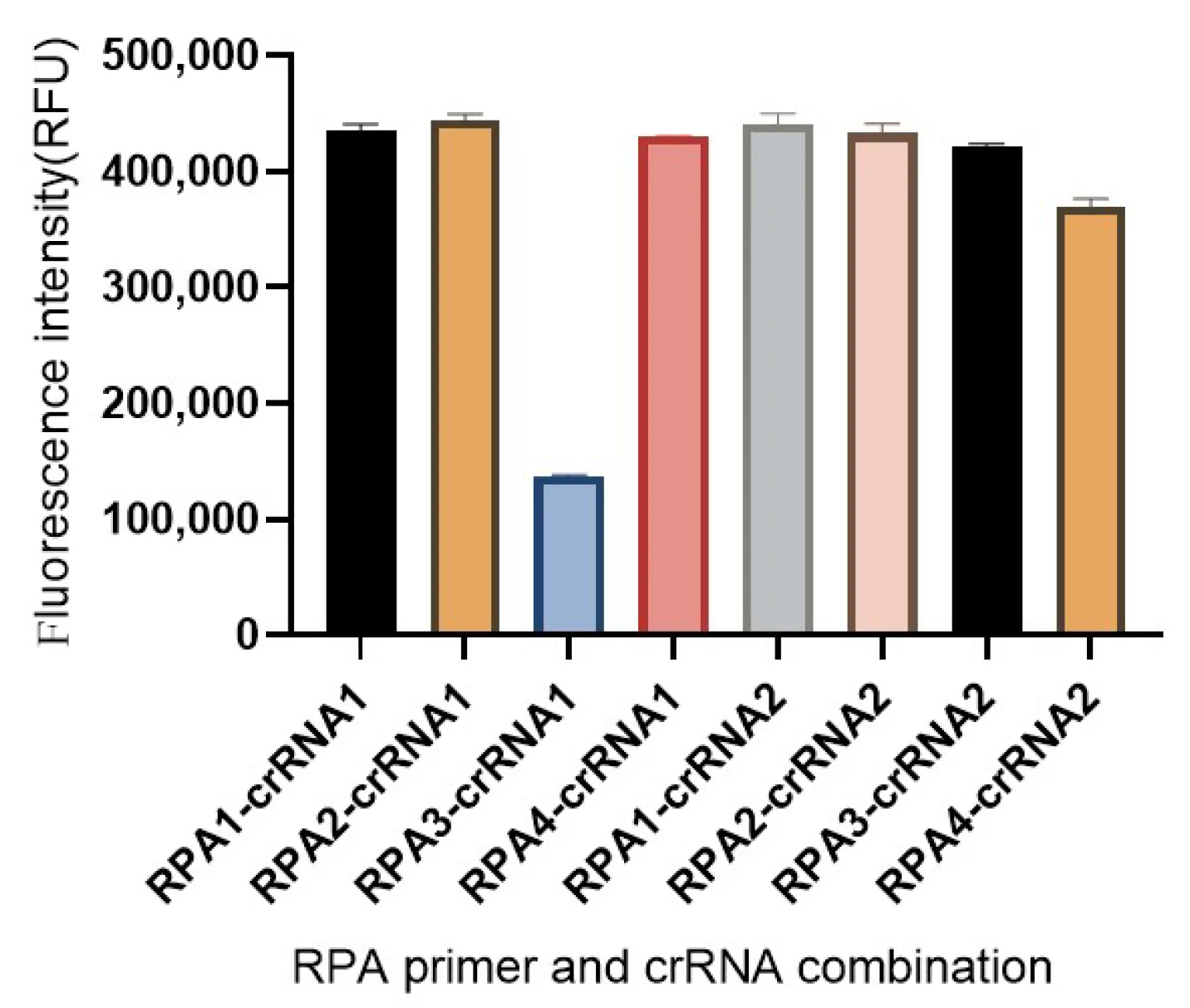
Endpoint fluorescence intensity analysis of RPA primer and crRNA combinations. Bars represent the mean of three replicates, and error bars represent the standard deviation (SD).

The four RPA products were combined with crRNA1 or crRNA2, and fluorescence signals were compared among the eight combinations. As shown in Figure 2 and Figure 3, fluorescence intensity differed among the combinations, indicating that both RPA efficiency and crRNA recognition efficiency affected the final CRISPR/Cas12a detection performance. Among them, RPA2-crRNA1 showed the highest mean endpoint fluorescence intensity (443,934.00 ± 5488.36), followed by RPA1-crRNA2 (440,576.67 ± 9287.69), RPA1-crRNA1 (435,067.67 ± 5709.31), RPA2-crRNA2 (432,610.00 ± 8242.70), and RPA4-crRNA1 (430,049.00 ± 315.40). The RPA3-crRNA1 combination showed a relatively weak fluorescence signal (137,370.00 ± 640.94). One-way ANOVA showed that endpoint fluorescence intensity differed significantly among the eight combinations (*p* < 0.0001). Based on visual fluorescence, endpoint fluorescence intensity, and RPA performance, RPA2-crRNA1 was selected as the optimal combination for subsequent optimization.

### 3.3. Optimization of crRNA and Cas12a Protein Concentrations

**Figure 4:**
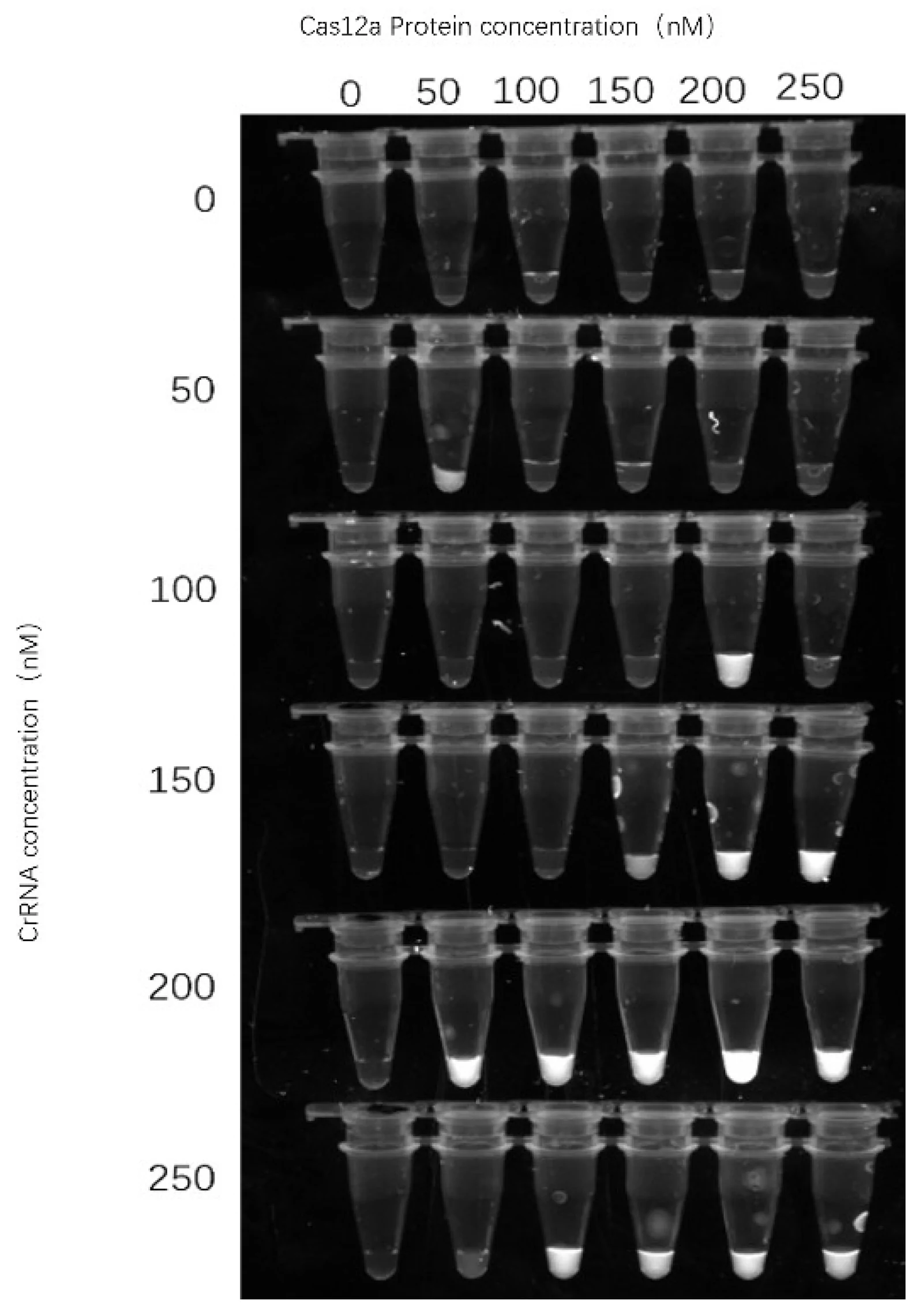
Orthogonal analysis of crRNA and Cas12a protein concentrations.

**Figure 5:**
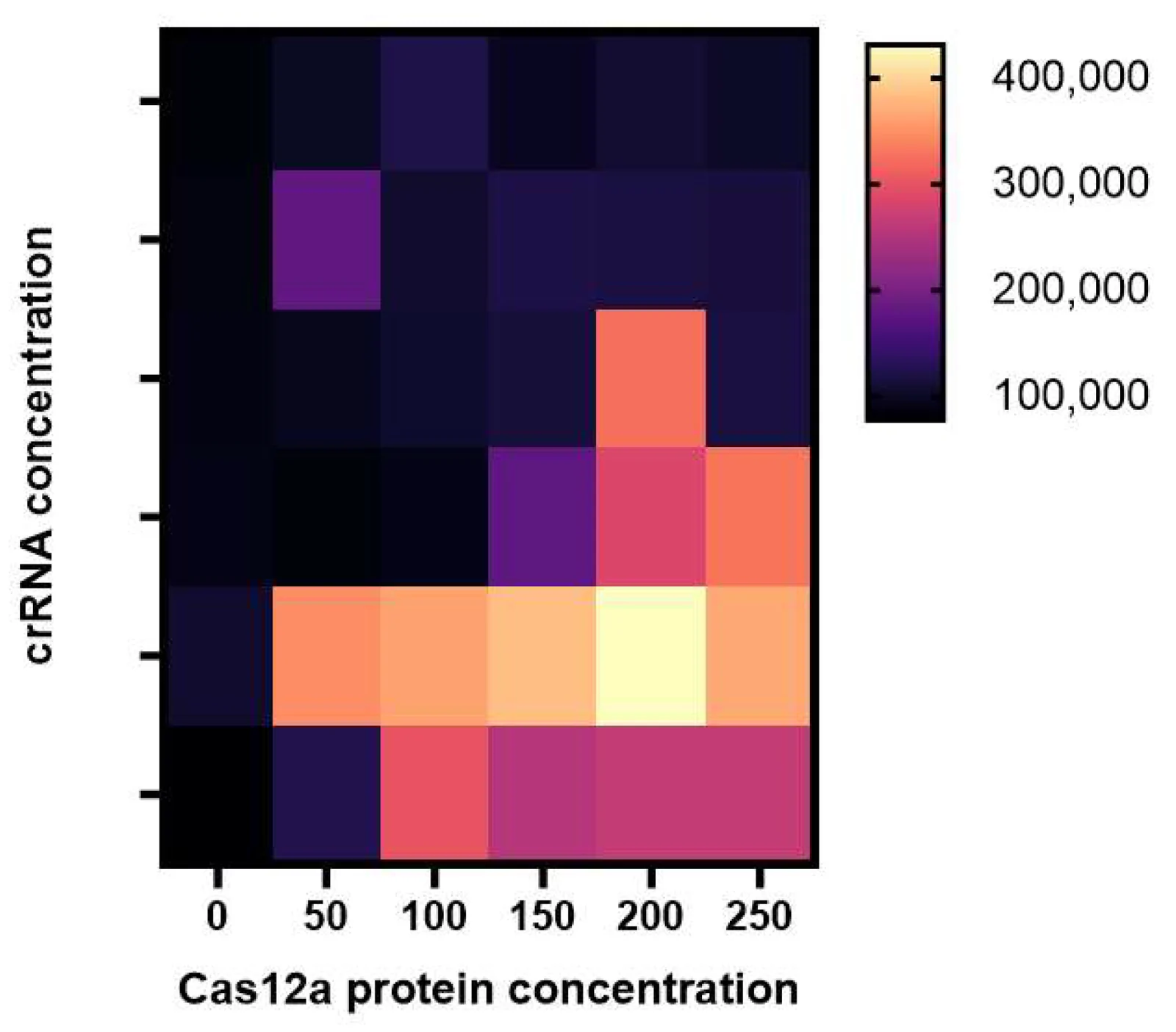
Heatmap of fluorescence values from the orthogonal experiment.

When the concentration of Cas12a protein or crRNA was 0 nM, almost no fluorescence signal was detected. As shown in Figure 4 and Figure 5, the fluorescence signal generally increased with increasing concentrations of Cas12a protein and crRNA. Considering fluorescence intensity, background signal, and reagent cost, 200 nM Cas12a protein and 200 nM crRNA were selected for subsequent experiments.

### 3.4. Optimization of ssDNA Reporter Concentration

**Figure 6:**
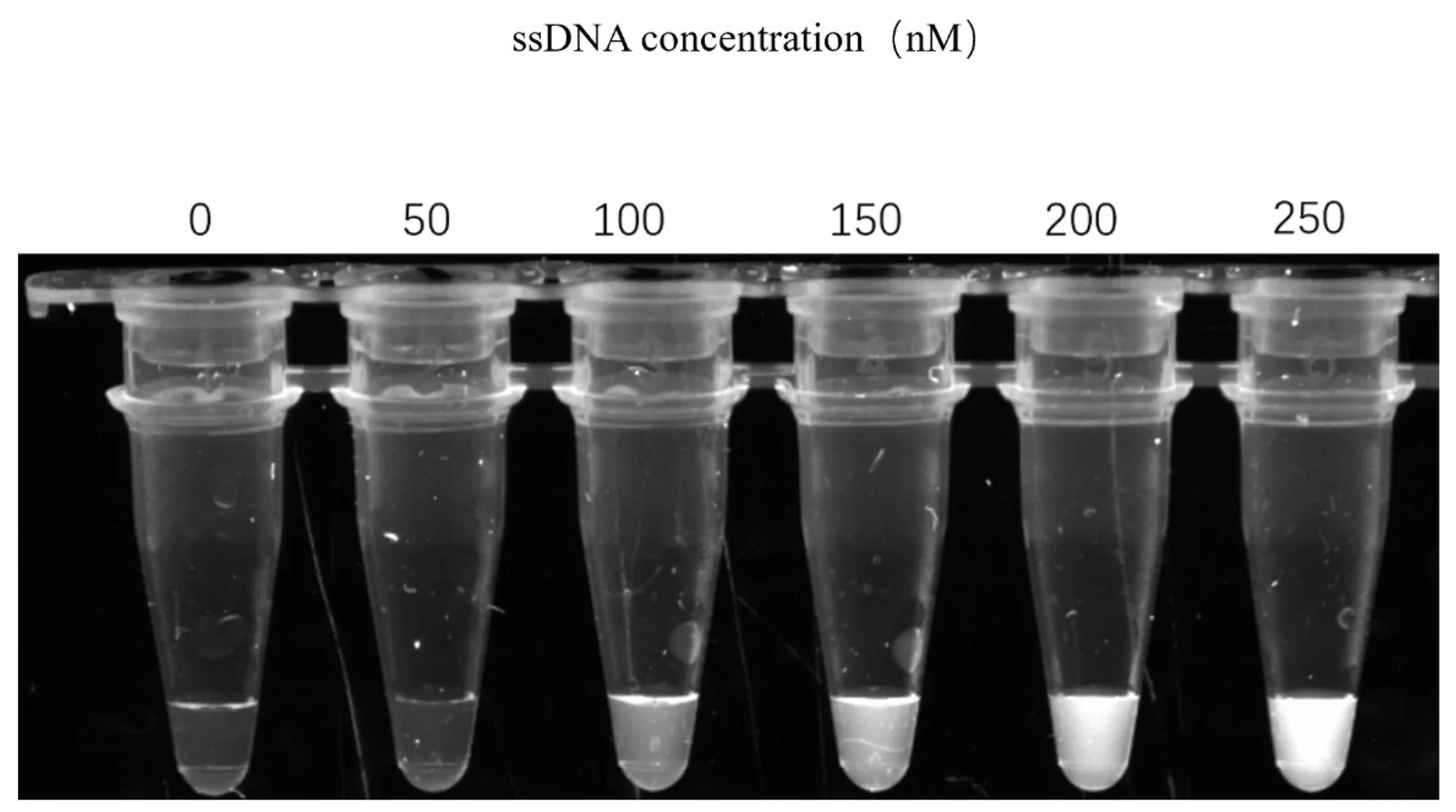
Optimization image of ssDNA reporter concentration.

As shown in Figure 6, no obvious fluorescence signal was observed when the ssDNA reporter concentration was 0 nM. The fluorescence signal increased as the reporter concentration increased. Considering that a higher reporter concentration may increase reagent cost, 250 nM was selected as the working concentration of the ssDNA fluorescent reporter for subsequent assays.

### 3.5. Analytical Sensitivity Results

**Figure 7:**
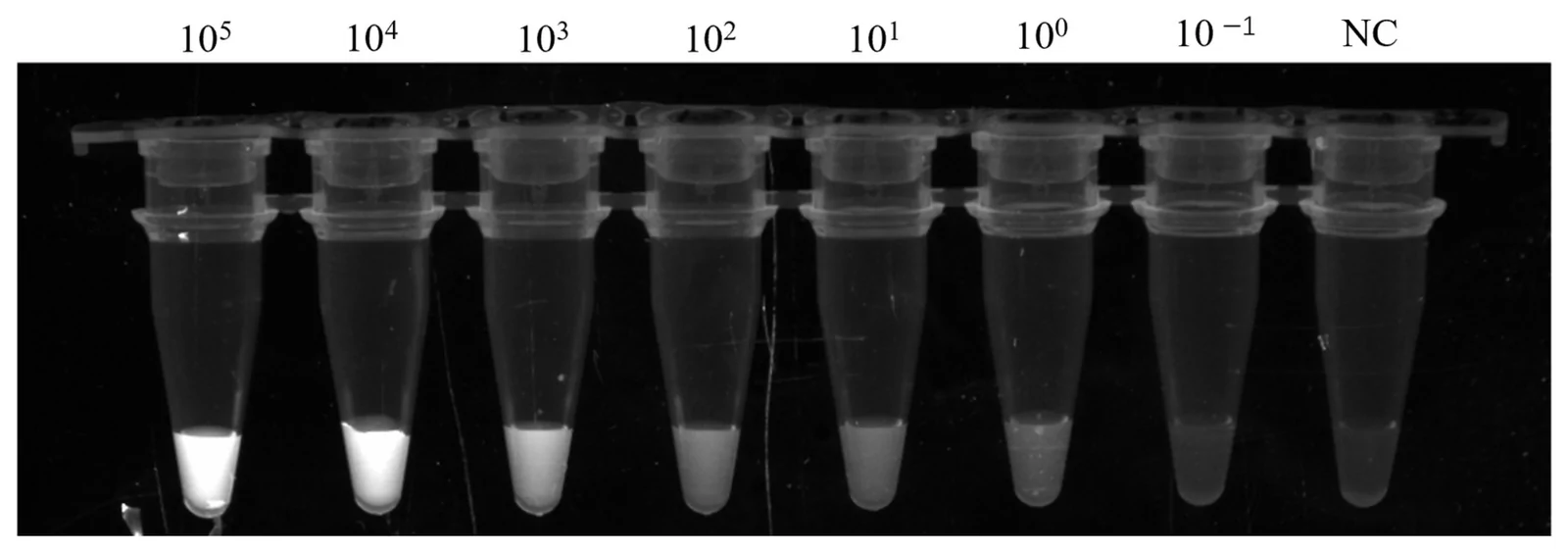
Sensitivity test results of the RPA-CRISPR/Cas12a assay.

**Figure 8:**
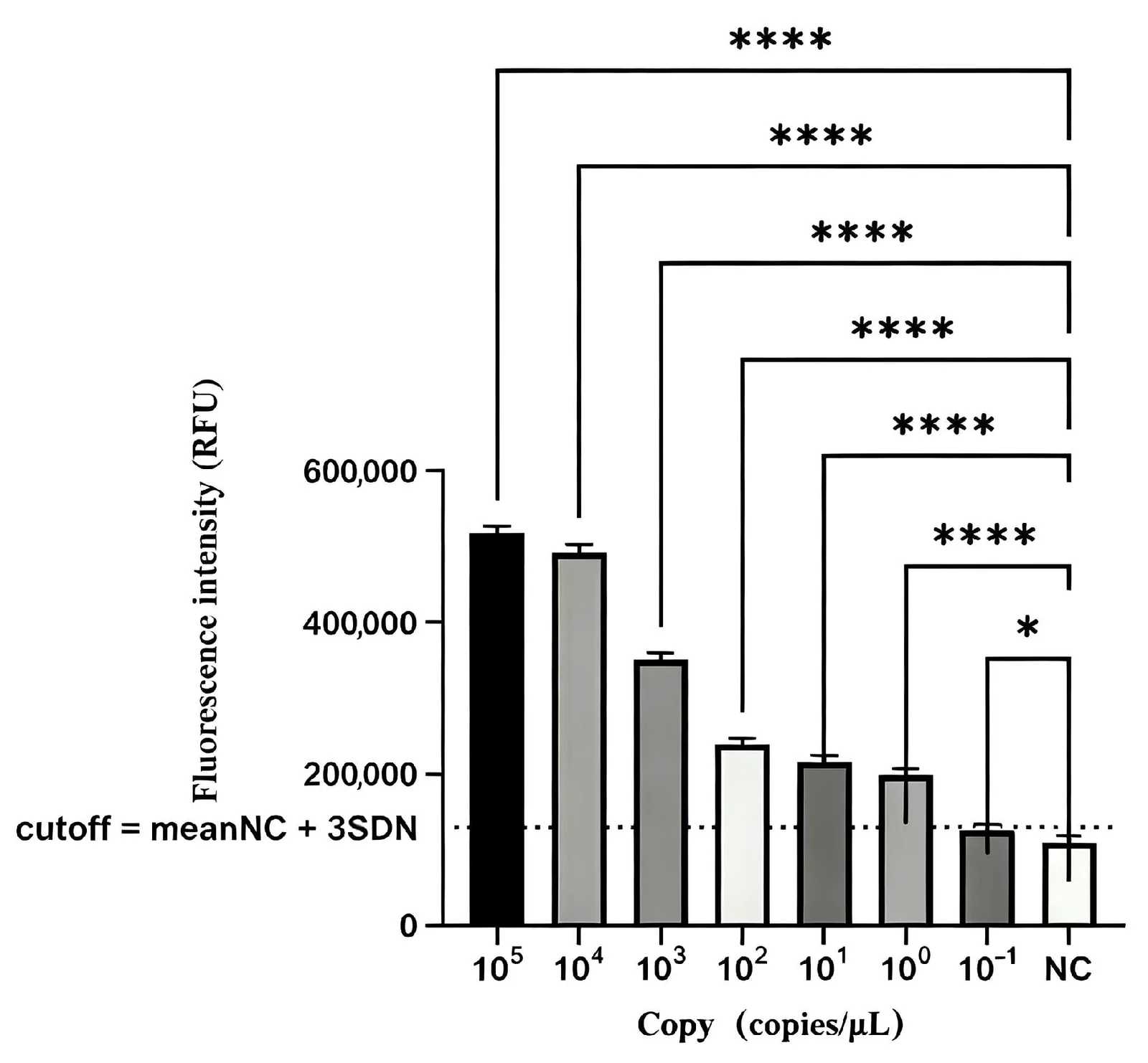
Fluorescence intensity analysis for the sensitivity test. Bars represent the mean of three replicates, and error bars represent the standard deviation (SD). NC, negative control. The dashed line indicates the positive cutoff, calculated as the mean fluorescence value of NC plus 3SD. The lowest template concentration exceeding the cutoff and producing stable visual fluorescence was defined as the limit of detection.

As shown in Figure 7, fluorescence intensity decreased gradually with decreasing template concentration. Strong fluorescence was observed at higher template concentrations, whereas the signal became weaker at lower concentrations. The 2.47 × 10^−1^ copies/μL group was close to the negative control. Based on visual interpretation and endpoint fluorescence analysis (Figure 8), 2.47 × 10^0^ copies/μL produced visible fluorescence and exceeded the positive cutoff. Although the fluorescence value of the 2.47 × 10^−1^ copies/μL group was slightly higher than that of the negative control, the signal was weak and did not stably meet the positive criterion. Therefore, the limit of detection of the established RPA-CRISPR/Cas12a assay was determined to be 2.47 × 10^0^ copies/μL.

### 3.6. Specificity Evaluation Results

**Figure 9:**
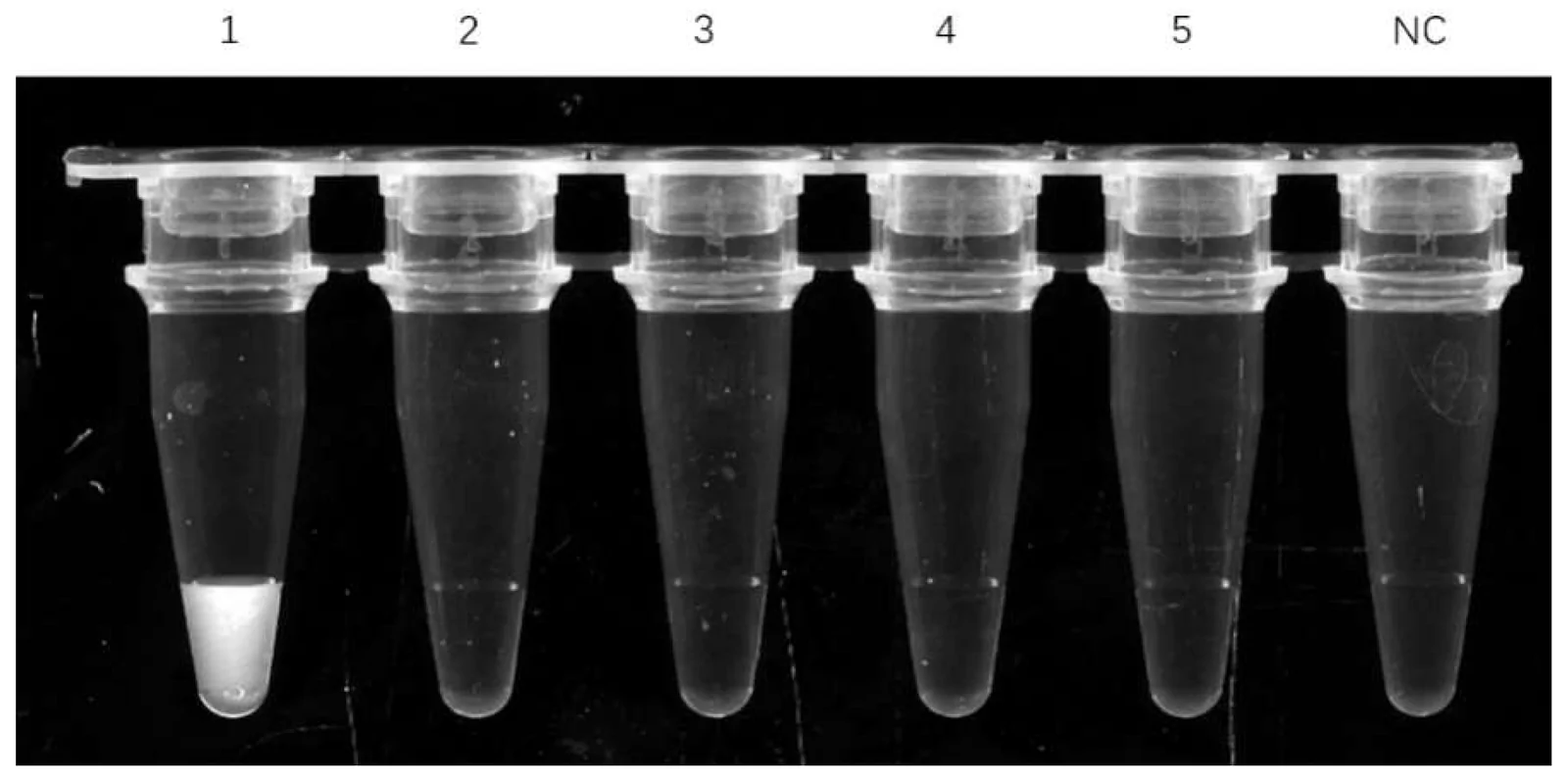
Specificity test results of the RPA-CRISPR/Cas12a detection system. 1, *Brucella*; 2, *Mycoplasma capricolum* subsp. *capripneumoniae*; 3, *Chlamydia abortus*; 4, *Chlamydia psittaci*; 5, *Salmonella enterica* serovar Typhimurium; NC, negative control.

The optimized assay was used to detect *Brucella* and four non-target pathogens. As shown in Figure 9, only the Brucella-positive template produced a positive fluorescence signal, whereas *Mycoplasma capricolum* subsp. *capripneumoniae*, *Chlamydia abortus*, *Chlamydia psittaci*, *Salmonella enterica* serovar Typhimurium, and the negative control showed no positive fluorescence. These results indicate that the established RPA-CRISPR/Cas12a system had good specificity under the tested conditions.

### 3.7. Clinical Sample Validation

Among the 56 clinical nasal swab samples, qPCR identified 22 positive and 34 negative samples. As shown in Table 5, the established RPA-CRISPR/Cas12a assay identified 20 positive and 36 negative samples. All 20 RPA-CRISPR/Cas12a-positive samples were also qPCR-positive, and no qPCR-negative sample was positive by the RPA-CRISPR/Cas12a assay. Two qPCR-positive samples were negative by the RPA-CRISPR/Cas12a assay and were considered qPCR-positive/RPA-negative discordant results. Compared with qPCR, the positive percent agreement, negative percent agreement, overall percent agreement, positive predictive value, and negative predictive value were 90.91% (20/22; 95% CI: 70.84–98.88%), 100.00% (34/34; 95% CI: 89.72–100.00%), 96.43% (54/56; 95% CI: 87.69–99.56%), 100.00% (20/20; 95% CI: 83.16–100.00%), and 94.44% (34/36; 95% CI: 81.34–99.32%), respectively. Cohen’s kappa value was 0.924 (95% CI: 0.805–1.000).

## 4. Discussion

Brucellosis is an important zoonotic disease caused by bacteria of the genus *Brucella*. It can result in abortion, stillbirth, infertility, decreased milk production, and substantial economic losses in livestock, particularly in cattle, sheep, and goats. In addition, brucellosis poses a potential occupational risk to veterinarians, farmers, laboratory personnel, and other individuals who are frequently exposed to infected animals or contaminated materials [13,14]. For livestock farms and primary animal disease prevention and control institutions, early detection of infected animals and rapid screening of suspected clinical samples are essential for reducing pathogen transmission and supporting disease control. Compared with conventional PCR or qPCR assays, the RPA-CRISPR/Cas12a system has several advantages, including isothermal amplification, short reaction time, simple operation, and visual result interpretation. RPA enables rapid amplification of target fragments under relatively simple equipment conditions, while the Cas12a-crRNA complex provides an additional sequence-specific recognition step and generates fluorescence signals through trans-cleavage of ssDNA reporters [15]. Therefore, the *bcsp31*-targeted RPA-CRISPR/Cas12a assay established in this study may provide a practical molecular screening tool for *Brucella* spp. detection in primary veterinary laboratories and field surveillance settings.

In this study, the bcsp31 gene was selected as the detection target. The bcsp31 gene encodes the 31 kDa immunogenic protein BCSP31 and is one of the commonly used molecular targets for Brucella detection. Because this gene is relatively conserved within the genus Brucella, it is suitable for genus-level screening of *Brucella* spp. Several studies have used bcsp31 to establish nucleic acid detection methods for Brucella. Becker et al. [16] demonstrated the application value of the bcsp31 target in Brucella PCR detection, indicating that bcsp31 can serve as an effective target for molecular diagnosis. Shukla et al. [17] established a LAMP assay targeting the bcsp31 gene, which detected as low as 8 fg of Brucella DNA within 35 min, with a sensitivity of 90.91%, a specificity of 99.37%, and an accuracy of 98.60%. Liu et al. [18] developed a one-tube nested real-time PCR assay targeting bcsp31, with an analytical sensitivity of 100 fg/μL, approximately 100 times higher than that of conventional qPCR. In clinical sample detection, the sensitivity and specificity of that method were 98.6% and 100%, respectively. These studies indicate that bcsp31 is not only a conserved and commonly used target for Brucella detection but also suitable for the development of various sensitive nucleic acid detection methods, including PCR, qPCR, and LAMP. Therefore, the selection of bcsp31 as the target gene in the present RPA-CRISPR/Cas12a assay is supported by previous studies and has a clear methodological basis.

RPA-CRISPR/Cas12a-based nucleic acid detection has been increasingly applied to rapid and visual detection of different pathogens. Previous studies have shown that this strategy has the advantages of rapid amplification, sequence-specific CRISPR/Cas12a recognition, low equipment dependence, and visual result interpretation [19]. For example, this approach has been used for the detection of Toxoplasma gondii in animal samples [20] and for visual detection of plant pathogens such as Phytophthora sojae and Phytophthora root rot pathogens [21,22]. In addition to veterinary and animal disease detection, RPA-CRISPR/Cas12a-based assays have also been explored in other fields, including plant disease diagnosis and food safety-related pathogen detection. These studies indicate that RPA-CRISPR/Cas12a is a flexible platform with potential for on-site pathogen detection. Based on this technical background, the present study further applied this strategy to the visual detection of *Brucella* spp. in veterinary surveillance samples.

Several RPA-CRISPR/Cas12a-based assays have been reported for *Brucella* detection. Dang et al. [23] developed a CRISPR-Cas12a test strip package targeting the *bp26* gene for in-situ detection of *Brucella* from infected livestock, with lateral flow readout and a reported sensitivity of 10 copies/μL. Shen et al. [24] developed an RPA-CRISPR/Cas12a assay for *Brucella melitensis* based on the *omp31* target and established both fluorescence and lateral flow strip readouts, with reported detection limits of 1 copy/μL and 10 copies/μL, respectively. Deng et al. [25] reported a BCSP31-based RPA-CRISPR/Cas12a assay for *Brucella* detection and evaluated the assay using clinical blood samples. Compared with these studies, the present assay targets the conserved *bcsp31* gene and achieved an analytical detection limit of 2.47 × 10^0^ copies/μL using a pUC19-*bcsp31* plasmid template. In addition, the present study systematically screened RPA primer–crRNA combinations, optimized key CRISPR/Cas12a reaction components, and preliminarily evaluated diagnostic agreement with qPCR using 56 sheep nasal swab samples collected during routine surveillance. Therefore, the present work provides additional methodological evidence for a *bcsp31*-targeted visual RPA-CRISPR/Cas12a workflow and its potential application in routine sheep surveillance samples.

In the RPA-CRISPR/Cas12a detection system, the final fluorescence signal is influenced by both RPA efficiency and crRNA-guided Cas12a recognition efficiency. Although RPA has the advantages of isothermal amplification and rapid reaction, different primer pairs may show different amplification efficiencies because of differences in amplicon length, sequence structure, primer dimer formation, and non-specific amplification. In addition, the CRISPR/Cas12a reaction requires that the RPA product contains a suitable crRNA recognition sequence and an appropriate PAM-related region. Therefore, the intensity of the RPA electrophoresis band alone cannot fully represent the final detection performance of the RPA-CRISPR/Cas12a assay. In this study, four RPA primer pairs were first screened by agarose gel electrophoresis, and then the corresponding RPA products were further evaluated in combination with two crRNAs using fluorescence readout. Based on both amplification performance and fluorescence intensity, the RPA2-crRNA1 combination was selected as the optimal detection combination. This result suggests that, when establishing an RPA-CRISPR/Cas12a assay, the amplification efficiency of RPA, the recognition efficiency of crRNA, and the final fluorescence output should be comprehensively evaluated.

Cas12a protein, crRNA, and ssDNA fluorescent reporter are key components of the CRISPR/Cas12a detection system, and their concentrations directly affect fluorescence intensity, background signal, reagent consumption, and detection stability. In this study, the concentrations of Cas12a protein and crRNA were optimized using an orthogonal design, and 200 nM Cas12a protein and 200 nM crRNA were selected as the working concentrations. Subsequently, different concentrations of ssDNA fluorescent reporter were evaluated, and 250 nM was selected for subsequent experiments. These results indicate that appropriate optimization of key reaction components is important for improving fluorescence visualization and obtaining stable detection performance.

Analytical sensitivity is an important parameter for evaluating nucleic acid detection methods. In this study, the pUC19-*bcsp31* positive plasmid was used as the template for 10-fold serial dilution, and the fluorescence signal gradually decreased as the template concentration decreased. Based on the fluorescence cutoff and visual interpretation, the lowest detectable concentration of the established assay was determined to be 2.47 × 10^0^ copies/μL, indicating that the method can detect low-copy *Brucella* nucleic acid templates. Compared with conventional PCR, RPA does not require repeated temperature cycling and can amplify target sequences under mild isothermal conditions. Compared with qPCR, the RPA-CRISPR/Cas12a assay allows the amplified target to be further recognized by the Cas12a-crRNA complex, and the cleavage of ssDNA fluorescent reporters produces a visible fluorescence signal under blue or ultraviolet light. Therefore, this method reduces dependence on large real-time PCR instruments and may be more suitable for rapid preliminary screening in field or primary laboratory settings.

It should be noted that a statistically higher fluorescence value in a low-template-concentration group does not necessarily mean that this concentration can be regarded as a stable positive detection limit. For visual RPA-CRISPR/Cas12a detection, determination of the limit of detection should consider the fluorescence threshold, repeatability, and visual interpretation results together. Therefore, in this study, the positive cutoff was calculated as the mean fluorescence value of the negative control plus three standard deviations, and the lowest concentration that exceeded the cutoff and produced stable visual fluorescence was defined as the limit of detection. Compared with conventional PCR, in which results are mainly judged by observing electrophoretic bands, the RPA-CRISPR/Cas12a system includes an additional target sequence recognition step after amplification. This helps reduce the influence of non-specific amplification on result interpretation and improves the reliability of visual detection.

The specificity test showed that, under the optimized reaction conditions, only the *Brucella*-positive template produced a clear positive fluorescence signal, whereas nucleic acids from *Mycoplasma capricolum* subsp. *capripneumoniae*, *Chlamydia abortus*, *Chlamydia psittaci*, *Salmonella enterica* serovar Typhimurium, and the negative control did not produce positive reactions. These four non-target pathogens were selected because they are clinically relevant pathogens considered in the differential diagnosis of sheep abortion and because positive clinical materials were available in Ningxia during the study period. The results indicate that the established *bcsp31*-targeted RPA-CRISPR/Cas12a assay showed good specificity for *Brucella* spp. under the tested conditions and did not cross-react with the selected non-target pathogens. However, the specificity panel was still limited and did not include phylogenetically closer organisms or bacteria known to be associated with diagnostic cross-reactions in brucellosis, such as *Ochrobactrum* spp. and *Yersinia enterocolitica* O:9. Therefore, the specificity results should be interpreted as preliminary.

Because *bcsp31* is a conserved target within the genus *Brucella*, the assay established in this study is more suitable for genus-level screening of *Brucella* spp. and cannot be used alone to distinguish *Brucella melitensis*, *Brucella abortus*, or *Brucella suis*. It also cannot differentiate vaccine strains from wild-type strains. Therefore, this method should be regarded as a rapid preliminary screening tool rather than a species identification or strain-typing method. In the present study, no *Brucella* vaccine was administered to the sampled animals according to the available vaccination records; therefore, *Brucella* vaccine strains or vaccine-derived *Brucella* nucleic acids were not expected to affect the interpretation of the qPCR or RPA-CRISPR/Cas12a results. Nevertheless, vaccination history remains an important factor to consider when applying nucleic acid-based *Brucella* detection methods in field surveillance. For samples with positive results, further confirmation using qPCR, bacterial isolation, sequencing, serological testing, or epidemiological investigation may be required depending on the diagnostic purpose.

In this study, 56 sheep nasal swab samples collected during routine second-quarter brucellosis surveillance were used for preliminary clinical validation of the established RPA-CRISPR/Cas12a assay, and qPCR was used as the comparator assay for diagnostic agreement analysis. Nasal swabs were selected because they are non-invasive, rapid to collect, cause relatively little stress to animals, and can be collected from both male and female sheep. Notably, no qPCR-negative sample was positive by the RPA-CRISPR/Cas12a assay, indicating good negative agreement and no qPCR-negative sample was positive by the RPA-CRISPR/Cas12a assay under the tested sample conditions. However, two qPCR-positive samples were negative by the RPA-CRISPR/Cas12a assay, which may be related to low pathogen nucleic acid content, the complex matrix of nasal swab samples, sample preservation and transportation conditions, DNA extraction efficiency, or the presence of inhibitors affecting RPA. Because the clinical samples used in this study were obtained from routine surveillance rather than experimentally infected animals, the clinical validation mainly reflects the preliminary applicability of the assay in field surveillance samples. Further studies should include a larger number of samples from different regions, sample types, and infection stages, and systematic comparisons with qPCR, serological assays, or pathogen isolation where feasible should be conducted to more comprehensively evaluate the stability and applicability of this method in clinical screening and primary veterinary surveillance settings.

In summary, this study established a visual RPA-CRISPR/Cas12a assay targeting the *bcsp31* gene for rapid detection of *Brucella* spp. The assay has the advantages of isothermal amplification, short reaction time, and visual result interpretation, and may provide a useful technical approach for preliminary brucellosis screening in primary veterinary laboratories and field surveillance settings. However, several practical limitations should be considered. First, the number of clinical samples was limited, and larger-scale validation using different sample types and samples from different regions is still required. Second, the current workflow still requires nucleic acid extraction and tube opening after RPA to perform the CRISPR/Cas12a reaction, which may increase the risk of aerosol contamination. Third, although the assay can be visually observed under ultraviolet or blue light, quantitative fluorescence measurement still requires a fluorescence reader, and the interpretation of weakly positive or suspected samples requires further optimization by combining fluorescence thresholds, visual readout, and qPCR results. Fourth, Cas12a protein, crRNA, and fluorescent reporters increase reagent cost compared with simple isothermal amplification alone, and the liquid reagents used in the current assay generally require cold-chain storage. Future studies should focus on simplified sample preparation, closed-tube or one-pot reaction formats, lateral flow strip readouts, lyophilized reagent systems, and larger-scale clinical validation to improve the field applicability and stability of the assay.

## 5. Conclusions

In this study, a visual RPA-CRISPR/Cas12a assay targeting the *Brucella bcsp31* gene was established. RPA2-crRNA1 was selected as the optimal detection combination, and the optimized system contained 200 nM Cas12a protein, 200 nM crRNA, and 250 nM ssDNA fluorescent reporter. The assay detected as low as 2.47 × 10^0^ copies/μL of pUC19-*bcsp31* plasmid template and showed no cross-reactivity with the tested non-target pathogens. Preliminary clinical validation using 56 sheep nasal swab samples showed strong diagnostic agreement with qPCR. The established assay is rapid and visually interpretable and may serve as a potential tool for preliminary *Brucella* spp. screening in primary veterinary surveillance settings. However, larger-scale validation using different sample types and samples from different regions is still needed.

## Figures and Tables

**Table 1:** Main reagents used in this study.

Reagent	Manufacturer
DNA/RNA extraction kit	Tiangen Biotech (Beijing) Co., Ltd., Beijing, China
Universal DNA purification kit	Tiangen Biotech (Beijing) Co., Ltd., Beijing, China
DH5α competent cells	Tiangen Biotech (Beijing) Co., Ltd., Beijing, China
RPA nucleic acid amplification kit	Suzhou Keer Life Technology Co., Ltd., Suzhou, Jiangsu, China
Cas12a protein	Shanghai Tolo Biotech Co., Ltd., Shanghai, China
ssDNA fluorescent reporter	Shanghai Tolo Biotech Co., Ltd., Shanghai, China
Plasmid DNA extraction kit	Changsha Xinlizhihe Technology Co., Ltd., Changsha, Hunan, China

**Table 2:** Main instruments used in this study.

Instrument	Manufacturer
Autoclave	Shanghai Boxun Medical Biological Instrument Corp., Shanghai, China
Constant-temperature water bath	Changzhou Guohua Electric Appliance Co., Ltd., Changzhou, Jiangsu, China
Vertical clean bench	Nantong Jiacheng Instrument Co., Ltd., Nantong, Jiangsu, China
Mini vortex mixer	Jiangsu Tianli Medical Device Co., Ltd., Taizhou, Jiangsu, China
Mini centrifuge	Haimen Qilinbeier Instrument Manufacturing Co., Ltd., Haimen, Nantong, Jiangsu, China
High-speed centrifuge	Bio-Rad Laboratories, Hercules, CA, USA
Gel imaging system	Bio-Rad Laboratories, Hercules, CA, USA
Real-time PCR system	Sichuan Jielaimei Technology Co., Ltd., Chengdu, Sichuan, China
Electrophoresis apparatus	Beijing Liuyi Biotechnology Co., Ltd., Beijing, China

**Table 3:** RPA primer and crRNA sequences used in this study.

	Sequence (5′–3′)	Amplicon Size (bp)
RPA-F1	GTTGCCAATATCAATGGATCAAGTCGGGCG	215
RPA-R1	CGCTTGCCTTTCAGGTCTGCGGACCGATTTGA	
RPA-F2	GGCTCGGTTGCCAATATCAATGCGATCAAGT	303
RPA-R2	ATCGTCTTCCGTGAGGCCGTAGGCTTCAAGA	
RPA-F3	CCTATTGGGCCTATAACGGCACCGGCCTTTA	299
RPA-R3	GGCGTCCAGCGCACCATCTTTCAGCCTCTCG	
RPA-F4	GGTTGCCAATATCAATGGGATCAAGTCGGGC	372
RPA-R4	AAGAAATAGGCGTCCAGCGCACCATCTTTCA	
crRNA-1	AAUUUCUACUAAGUGUAGAUCGCAGUCAGACGUUGCCUAUUGG	—
crRNA-2	AAUUUCUACUAAGUGUAGAUCCCGGAAACGAUCCAUAUCGUUG	—

**Table 4:** CRISPR/Cas12a reaction system.

Component	Volume
10× HOLMES Buffer for Cas12a	2 μL
10 μM LbCas12a protein	0.5 μL
10 μM crRNA	0.5 μL
RPA product	3 μL
10 μM ssDNA fluorescent reporter	0.5 μL
Nuclease-free water	To 20 μL

**Table 5:** Comparison of the RPA-CRISPR/Cas12a assay and qPCR for clinical sample detection.

RPA-CRISPR/Cas12a Result	qPCR-Positive	qPCR-Negative	Total
Positive	20	0	20
Negative	2	34	36
Total	22	34	56

## Data Availability

The data supporting the findings of this study are included in the article. Additional de-identified data are available from the corresponding author upon reasonable request.
